# Evaluation of the Training Program to Train HIV Treatment Center Staff in Pakistan

**DOI:** 10.7759/cureus.61972

**Published:** 2024-06-08

**Authors:** Sharaf A Shah, Syed Hani Abidi, Altaf A Soomro, Nida Farooqui, Tehreem Ansari, Rafiq Khanani

**Affiliations:** 1 Infectious Disease, Bridge Consultant Foundation, Karachi, PAK; 2 Biomedical Sciences, Nazarbayev University School of Medicine, Astana, KAZ; 3 Biological and Biomedical Sciences, Aga Khan University Medical College, Karachi, PAK; 4 Infectious Diseases, Dow University Hospital, Dow University of Health Sciences, Karachi, PAK; 5 Medicine, Baqai Medical University, Karachi, PAK

**Keywords:** allied health personnel, healthcare professionals in pakistan, knowledge assessment, antiretroviral therapy and human immunodeficiency virus, skill training

## Abstract

Introduction

In Pakistan, HIV training programs, especially for health professionals working in HIV treatment centers, are limited. Consequently, there is little data about HIV awareness among physicians and allied health workers and how it may affect their care for people living with HIV (PLWH). Recently, the Global Fund to Fight AIDS, Tuberculosis, and Malaria (GFATM) grant Principal Recipient UNDP engaged an NGO experienced in HIV/AIDS training, on a competitive basis, to develop a training manual and conduct training of all categories of HIV treatment centers staff. The goal of this study was to assess the training program's influence on trainees' (both physicians and allied health staff) knowledge and abilities and describe its major lessons.

Methodology

This was a one-group pre-post test study, carried out between January 17 and February 22, 2023. The study was carried out in three phases. In the first phase, a team of experts developed an antiretroviral treatment (ART) training manual. In the second phase, 9- and three-day training workshops were conducted in six different cities of Pakistan, which were attended by physicians and allied health staff working in different HIV treatment centers across Pakistan. The workshops had plenary lectures, discussions, role plays, video cases, and case studies. In the third phase, a quiz, comprising multiple/best choice questions (MCQs/BCQs) and true and false questions, was administered before (pre) and after the workshop (post) to assess the impact of these training sessions in enhancing the level of HIV knowledge, especially related to ART. The workshop was attended by a total of 256 health workers from different cities in Pakistan. The participants had backgrounds in medical science, psychology, laboratory science, nursing, and computer science. Pre-and post-test responses were statistically analyzed to determine the impact of the training program on participant's knowledge. For this, the Shapiro-Wilk test was applied to test data normality, followed by the application of paired t-test or Wilcoxon Signed Rank Test for normally and non-normally distributed data, respectively. Finally, a chi-square test was applied to examine the significant (p<0.05) association between training workshops and improvement in the participant’s level of understanding of HIV. In all statistical tests, p<0.05 was considered significant.

Results

The results from our study showed that before the training session, both physicians and allied staff possessed limited knowledge about HIV-related domains. After the workshops, participants from all cities demonstrated a uniform enhancement of knowledge related to different HIV-related domains, evident from the improvement in post-test scores compared to pre-test scores (p<0.0001). The chi-square test showed a significant association between training workshops and improvement in the participant’s level of understanding about HIV (p-values for BCQ, MCQ, and true and false: 0.001, 0.0047, and 0.0024, respectively).

Conclusions

Pre- and post-test evaluation provides an objective, data-driven method for measuring the impact of educational interventions in improving healthcare workers' awareness about HIV. The results emphasize the role of continuous workshops and training programs in enhancing the knowledge and understanding of healthcare and allied health workers regarding HIV.

## Introduction

HIV prevalence in the general population of Pakistan is approximately 0.2% [1]. However, HIV exists as a concentrated epidemic in key population groups such as people who inject drugs, transgender sex workers, male sex workers, men who have sex with men, and female sex workers, where the prevalence is approximately 38.4%, 7.5%, 5.6%, 5.4%, and 2.2%, respectively [2,3]. Furthermore, the number of new cases has increased rapidly in the past 10 years, and several new outbreaks have occurred in Pakistan in recent years, including the 2019 HIV outbreak in Larkana, where more than 1,000 children were found infected with HIV [2-9].

Globally, the field of HIV treatment and care has been experiencing significant growth, with the expansion of healthcare provider education and training being identified as a crucial factor contributing to the accelerated improvement in the delivery of high-quality care [10-13]. Several studies have shown that the implementation of intensive and interactive professional training workshops focused on HIV can lead to enhanced knowledge, attitudes, and willingness among healthcare professionals (HCPs) to deliver comprehensive care to individuals affected by HIV [14,15]. Here both physicians and allied health workers play a crucial role in enhancing the well-being of people living with HIV (PLWH) and their families through the provision of high-quality physical care and emotional support [16]. Studies have found that quantitative surveys and patient records were the most used assessment methods, providing an outcome of learning and downstream results in HCPs; however, there still are gaps in the literature regarding objective information on trainee behavior change [17]. Most studies use pre- and post-training tests to assess trainee learning, likely because these tests can be conducted easily after training sessions without additional follow-up; however, they are used only for testing factual knowledge [18]. However, there is limited information regarding the level of HIV knowledge possessed by both physicians and allied health workers in different regions globally, especially Pakistan, and its potential impact on their provision of care for PLWH.

To strategize and develop the skills, abilities, and resources of Pakistan’s healthcare system and to provide better HIV care, sustainable processes need to be formed based on partnerships between local academic institutions and antiretroviral treatment (ART) centers [19]. In Pakistan, national and provincial AIDS control programs have established more than 51 HIV treatment centers throughout the country [20]. However, comprehensive HIV training programs, especially for the HIV treatment center staff, are limited, and consequently, information regarding the level of HIV knowledge possessed by both physicians and allied health workers is scanty. Additionally, very little is known about how different education interventions contribute to the improvement of the level of HIV knowledge in Pakistani healthcare workers. Therefore, comprehensive training of all categories of HIV treatment center staff is necessary to provide high-quality and user-friendly services to PLWH. Recently, supported by the Global Fund to Fight AIDS, Tuberculosis, and Malaria (GFATM), and Bridge Consultants Foundation, an NGO experienced in HIV/AIDS training developed a training manual and conducted training of all categories of HIV treatment center staff.

The purpose of this study was to evaluate the impact of the training program on enhancing the knowledge and skills of trainees (both physicians and allied health workers) and document the key lessons learned from this training program.

## Materials and methods

Training manual and workshops

This was a one-group pre-post test study, comprising development and implementation phases. In the development phase, a three-member team of experts, comprising an infectious disease specialist, a microbiologist/laboratory expert, and a public health physician, all with substantial experience in the HIV domain, developed the ART training manual.

In the implementation phase, a team of trainers conducted training sessions for staff from 51 ART centers across Pakistan. For logistic reasons, a total of six cities (Faisalabad, Islamabad, Karachi, Lahore, Multan, and Peshawar) were selected as training locations, and representative staff from ART centers were invited. 

Three full-day training workshops were offered to all participants, between January 17 and February 22, 2023, which were attended by a total of 256 healthcare and allied health workers from different cities in Pakistan. The participants had backgrounds in medical science, psychology, laboratory science, nursing, and computer science.

In each workshop, different HIV experts (physicians, scientists, and public health specialists) delivered comprehensive sessions on different HIV knowledge domains, including the ART training manual (see Appendix; Table 4). The workshops had plenary lectures, discussions, role plays, video cases, and case studies.

Pre- and post-test quizzes and participant feedback

A quiz (available at https://bityl.co/QFED), comprising multiple choice questions (MCQs), best choice questions (BCQs), and true and false questions, was administered before (pre) and right after the workshop (post) to assess the impact of these training sessions in enhancing the level of HIV knowledge, especially related to ART. At the end of the workshop, a feedback form was administered to each participant to evaluate the participant’s experience/opinions about the workshop and the questions (see Appendix; Table 5). The study data was analyzed between March 1 and July 15, 2023. The data was collected after obtaining written informed consent from the participants. The study was approved by the Ethics Review Committee of the Bridge Consultant Foundation.

Statistical analysis

The pre- and post-test responses were statistically analyzed to determine the impact of the training program on participants' knowledge. For this, the Shapiro-Wilk test was applied to test data normality, followed by the application of paired t-test or Wilcoxon Signed Rank test, for normally (physicians test results) and non-normally distributed (allied health worker's test results) data, respectively, to examine the improvement in participants tests scores after the workshop (compared to the pre-test scores). Finally, a chi-square test was applied to examine the significant association between training workshops and improvement in the participant’s level of understanding of HIV. In all statistical tests, a p-value less than 0.05 was considered significant. All analyses were performed using GraphPad Prism version 9 for MS Windows.

## Results

Training outcomes 

**Figure 1:**
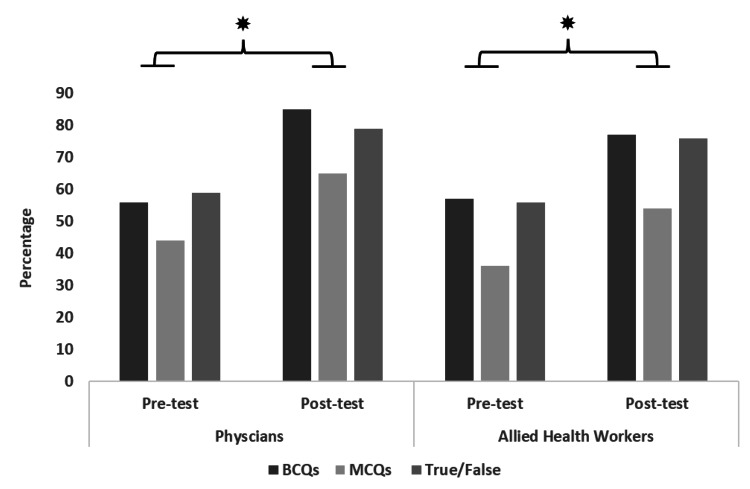
Results for pre- and post-test quizzes The bars show the percent change in correct responses before and after the workshop. The asterisks above the bar chart show a statistically significant difference (p<0.05). MCQs, multiple choice questions; BCQs, best choice questions

Overall, the pre-test results from all workshops showed that physicians were able to answer 56%, 44%, and 59% of BCQs, MCQs, and true/false questions correctly before the workshop, while after the workshop they were able to answer 88%, 65%, and 79% of BCQs, MCQs, and true/false questions correctly (Figure 1). Similarly, the pre-test results for other staff showed that they were able to 57%, 36%, and 56% of BCQs, MCQs, and true/false questions correctly before the workshop, while after the workshop they were able to answer 77%, 54%, and 76% of BCQs, MCQs, and true/false questions correctly (Figure 1).

The t-test or Wilcoxon signed rank test for pre- and post-workshop scores showed that participants from all cities demonstrated a uniform enhancement of knowledge related to different HIV-related domains, evident from the improvement in post-test scores compared to pre-test scores (p<0.0001). The chi-square test also showed a significant association between training workshops and improvement in the participant’s level of understanding about HIV (p-values for BCQ, MCQ, and true or false: 0.001, 0.0047, and 0.0024, respectively). 

The results from different cities also showed a significant improvement in the level of knowledge about HIV in both physicians and allied health workers post-workshop, where the most significant improvement was observed in both physicians and allied health workers from Peshawar (Table 1). For all cities, the chi-square test showed a significant association between training workshops and improvement in the participant’s level of understanding of HIV (p=0.001-0.004).

**Table 1:** City-wise results for pre- and post-test quizzes The table shows the city-wise percent change in correct responses before and after the workshop. The last columns show statistically significant differences (p-value) before and after the workshop. MCQs, multiple choice questions; BCQs, best choice questions

Cities	Type of questions	Physicians		Allied health workers		P-value
		Pre-test (%)	Post-test (%)	Pre-test (%)	Post-test (%)	
Faisalabad	BCQs	65	90	62	82	<0.05
	MCQs	55	68	49	63	
	True/false	65	88	67	78	
Islamabad	BCQs	36	90	67	83	
	MCQs	26	60	47	63	
	True/false	41	73	62	81	
Karachi (three workshops)	BCQs	52-59	72-88	26-59	59-77	
	MCQs	38-46	54-68	18-38	41-55	
	True/false	53-59	73-76	44-53	68-75	
Lahore	BCQs	48	90	66	84	
	MCQs	40	71	42	57	
	True/false	65	75	68	81	
Multan	BCQs	56	95	64	83	
	MCQs	52	66	38	53	
	True/false	61	79	64	79	
Quetta	BCQs	65	95	54	73	
	MCQs	42	59	35	54	
	True/false	69	81	51	72	
Peshawar	BCQs	66	95	59	85	
	MCQs	50	75	38	60	
	True/false	64	91	59	83	

Participant feedback

Up to 64% of the participants who responded to the feedback surveys rated the workshop as excellent, while up to 61% of the participants found the trainers knowledgeable and the workshop course content useful (Table 2). Overall, 62-88% felt that their expectations from the workshop were met to a greater extent (Table 2).

**Table 2:** Participants’ feedback on the workshop Table shows the expectations from the workshop given on the feedback survey forms

Questions	Excellent	Very good		Good	Fair		Poor
Rating of the three-day training workshop	13-64%	13-34%		18-25%	0%		0%
Trainers’ knowledge of the subject	33-61%	23-35%		3-21%	0%		0%
Training material of the workshop including PowerPoint slides, training manual, and handouts	55-61%	18-38%		2-18%	0%		3%
	To great extent		To some extent			Did not meet the expectation	
Workshop met the expectations	62-86%		11-31%			2-7%	

Lessons learned

The key lessons learned from the training sessions/workshops are summarized in Table 3.

**Table 3:** Key lessons learned from the training sessions/workshops

Key observations	Details
Pre-workshop knowledge gap	Prior to the commencement of the workshops, a noticeable lack of HIV-related knowledge was observed among all categories of staff working in ART centers.
The efficacy of the workshops and training	The results showed that the training sessions were effective in enhancing HIV-related knowledge in both physicians and other healthcare personnel. Overall, there was a significant association between the training workshops and the enhancement of participants’ comprehension of HIV.
Enhancement of knowledge across all categories of staff in all cities of Pakistan	The findings from all cities demonstrated a uniform enhancement in HIV knowledge among physicians and allied health workers following the training, highlighting the impact of the training workshops in improvement in HIV-related knowledge domains.
Significance of continuous training	The results emphasize the significant role of continuous workshops and training programs, offered to both physicians and allied health workers, in enhancing the knowledge regarding HIV.

## Discussion

The objective of the current study was to assess the impact of the training program in enhancing the HIV-related knowledge of the physicians and allied health staff working in different HIV treatment centers across Pakistan. The pre-assessment results showed that both physicians and allied staff possessed limited knowledge about HIV-related domains. After the workshops, participants from all cities demonstrated a uniform enhancement of knowledge related to different HIV-related domains (p<0.0001).

Assessment of behavior change after training programs helps in determining whether the objectives of the training interventions have been achieved; however, very few studies (a total of 30) have focused on HIV, showing surveys and interviews as a common method used for assessing behavioral changes in trainees [21]. The results from our study showed that prior to the training session, both physicians and allied staff possessed limited HIV-related knowledge domains. Our findings were in line with other published studies [22-25]. For example, a study from India showed that 85% of nurses did not adhere to the universal safety protocol, while 70% held the belief that HIV positivity and AIDS were interchangeable concepts [15]. Similarly, 13.5% of physicians were found to be unaware of the transmission of HIV through blood [15]. Another study from Madagascar showed that approximately 75% of the healthcare personnel, including physicians, midwives, nurses, medical students, and nursing auxiliaries, lacked essential knowledge about HIV transmission, testing, and management. For example, approximately 66% of HCPs indicated that they had never provided recommendations to patients regarding HIV testing, while 79% expressed concerns about their susceptibility to contracting HIV, primarily due to potential occupational exposure [26]. These findings identify an immense need to offer training programs to HIV physicians and allied health staff, especially those working in different HIV treatments and interacting with PLWH daily [11-16].

Using indicators from standard guidelines is another way to assess training programs such as TB control programs, where case detection and treatment success rate are the outcome indicators used for training programs [27]. However, for HIV training programs, HIV testing rate and proportion of patients with undetectable viral loads were used as outcome indicators. This indicators-based evaluation model, known as the goal-based Kirkpatrick model, which was developed in the 1960s, is still the most used evaluation framework and shaped the foundation for other frameworks [28]. Based on this need, we developed an HIV training manual and standardized evaluation framework while also offering training workshops across different cities of Pakistan. Our results showed that the workshops were effective in addressing the knowledge deficit and significantly improving (p<0.001) the awareness related to HIV knowledge domains among healthcare practitioners and allied health workers. Although the data on such educational interventions from Pakistan is limited, our findings are supported by a recent educational intervention study from Lahore, Pakistan showing a significant improvement in the level of HIV knowledge domains such as transmission, counseling, care, and interaction, after the educational intervention [29].

We anticipate certain limitations of our study. For example, information regarding different HIV domains was assessed; however, objective information on trainee behavior change and improvement in HIV care delivery could not be evaluated. Follow-up studies assessing the improvement in training behavior and HIV care delivery could provide valuable information about the long-term efficacy of such training programs. 

## Conclusions

In conclusion, the study indicates that the comprehensive training program, offered in the form of workshops, successfully bridged the knowledge gap related to HIV among healthcare practitioners and allied health workers. The positive impact of these workshops, evident from a significant improvement in post-test scores compared to pre-test (p<0.001), suggests that continuous and comprehensive HIV-related training is imperative for enhancing and sustaining the knowledge base of HCPs serving in HIV treatment centers throughout Pakistan.

Given the dynamic nature of HIV treatment and care, regular training and updates are necessary to ensure that healthcare practitioners, including physicians and allied health workers, are equipped with up-to-date information and best practices in managing HIV. Therefore, healthcare authorities and organizations should prioritize ongoing HIV-related training programs to ensure that healthcare practitioners remain well-informed and adept in providing optimal care to individuals living with HIV in Pakistan.

## Appendices

**Table 4:** HIV knowledge domain covered in each workshop STIs, sexually transmitted infections; PLHIV, people living with HIV; ART, antiretroviral treatment; PWIDs, people who inject drugs; PPTCT, parent-to-child transmission; VMMC, voluntary medical male circumcision; IRIS, immunoreconstitution inflammatory syndrome

Basic science	Clinical progression and pathogenesis
What are HIV and AIDS? Structure of the HIV virus. Modes of transmission. Natural history of HIV transmission	Seroconversion. Acute retroviral syndrome. Asymptomatic HIV infection. Symptomatic HIV infection (AIDS). Some common opportunistic infections in HIV/AIDS patients. HIV care continuum
HIV epidemiology	HIV counseling
Epidemiology of HIV/AIDS. Estimated PHIV, new HIV infections, and AIDS-related deaths, 1990-2021. Epidemiology of HIV/AIDS HIV prevalence among key populations, 2005-recent HIV prevalence among key populations, 2016-recent HIV/AIDS situation in Asia HIV/AIDS in Pakistan. UNAIDS fast-track strategy to end AIDS by 2030	Definition of counseling. Difference between counseling and education. Characteristics of a counselor. Characteristics of counseling session. Key principles of counseling. The steps that the counselor should follow. Purpose of HIV counseling. Pre- and post-test counseling including positive and negative patients. How to disclose HIV status to partner (sexual partner). Counseling for the prevention of HIV transmission. Counseling for ART adherence. How to counsel patients for ensuring follow-up counseling for specific groups
Providing user-friendly, high-quality HIV/AIDS prevention, diagnosis, treatment, and care and support services to key populations	HIV/AIDS prevention evidence-based recommendations
Define terms, key populations, and vulnerable populations and understand the behaviors of key populations. Define stigma and discrimination and how stigma undermines the health of key populations. Challenges faced by key populations in accessing healthcare services. Provide user-friendly services to key populations according to WHO recommendations. Ensure the confidentiality and privacy of the client. Understand why it is necessary to provide services under one roof. Know community-based HIV prevention services for key populations. Coordinate with NGOs and CBOs working with key populations	Evidence-based recommended strategies for HIV/AIDS prevention. How to prevent sexual transmission of HIV. Role of HIV testing and counseling in the prevention of HIV transmission. ART as an HIV prevention tool. Role of harm reduction program in the prevention of HIV transmission among PWIDs. Role of prevention of PPTCT of HIV. Role of VMMC. Role of safe injection practices, screening of blood before transfusion, and infection control in the prevention of HIV transmission
Comprehensive history-taking and focused clinical examination of HIV/AIDS patient	Video on clinical examination
How to assess the risk of the client for HIV and current health status. How we may take comprehensive history ensuring privacy and confidentiality in a non-judgmental manner. Conduct physical, genital, and rectal examinations including documentation of vital signs, height, weight, and systemic examination. Perform vaginal examination of women with vaginal speculum and conduct pap test. Perform rectal examination of men and women with a proctoscope and collect specimens for diagnostic test	Signs and symptoms of STIs among men and women. In the session, a video was displayed on signs and STIs among male and female patients
Role play on history-taking skill	HIV testing and diagnosis
What are good history-taking skills? A role play was demonstrated in front of participants to show them what good history-taking skills are and what are the common mistakes we make during history taking of a vulnerable person. Trainees among the participants take part in the role play.	Characteristic of HIV tests used in Pakistan approved by WHO. Rationale for HIV screening, confirmatory and molecular (PCR) tests. Guiding principles of HIV testing include 5Cs: consent, confidentiality, counseling, correct test results, connection to the care, and support services. WHO recommended HIV diagnosis, and three test strategies on rapid testing kits. Screening and conformity tests, including sensitivity and specificity of WHO-recommended HIV testing kits. Voluntary HIV confidential counseling and testing, care provider-initiated HIV testing and counseling, community-based HIV testing and counseling, and self-testing. Merits and demerits of different approaches. NAT and Viral load PCR tests, for diagnosis and monitoring and CD4 count, direct test, indirect test, and reference ranges for monitoring.
Clinical presentation of HIV	Antiretroviral therapy
Define HIV and AIDS. Identify the clinical presentation of HIV, elite responders, and IRIS. Describe and identify the clinical spectrum of HIV and categorize the disease according to the WHO staging in children and adults. Enlist differential diagnoses in patients presenting with symptoms involving different systems. The session was followed by the question and answer session	Define important terms used in ART. Providing ART to patients presenting at the ART center according to consolidated guidelines and monitoring patients on ART following recomposed protocol. Describe antiretroviral drugs, classification, and mechanism of action and resistance, and adverse effects of ARVs. Prescribe ART in adults, adolescents, children, and pregnant and lactating females
Live demonstration of HIV/HBV/HCV/syphilis serological tests	HIV co-infections and opportunistic infections: screening/clinical presentation/diagnosis/management/prevention
Alere HIV Combo - Early Detect Alere HIV – ½ Determine Unigold SD Bioline ½ 3.0 (Abbott) HBV. Determine HCV. Determine Syphilis TP (Abbott)	Screening of PLHIVs for opportunistic and co-infections. Diagnosing and treatment of viral hepatitis including hepatitis B, hepatitis C, and hepatitis D. Diagnosing, prevention, and treatment of AIDS-defining illnesses. Screening, prevention, diagnosing, and treatment of co-infections and opportunistic infections in PLHIV and CLHIV. Prescribe important vaccination
Therapy adherence; clinical presentation of HIV	TB HIV co-infection
Define adherence and describe the importance of adherence and the consequences of non-adherence. Identification of potential barriers to starting ART. Barriers to adherence. Monitoring adherence. Discuss strategies to promote adherence success. Discuss strategies to track lost to follow-up and reengage them. Basic strategies to re-initiate ART in lost to follow-up patients. Explain the link between adherence and resilience	Why it is important to learn about TB-HIV co-infection. How TB spreads. Natural history of TB infection and TB disease. How to deal with TB in PLHIV the three “S.” Intensify case finding in PLHIV. Identification of TB presumptive in PLHIV. Diagnosis of TB in PLHIV adults and children. How to ensure high-quality anti-tuberculosis treatment in PLHIV. Management of drug-sensitive TB. Management of drug resistance TB. Principle of management of TB in PLHIV. Special consideration in giving anti-TB drugs with ART. Time to start ART with ATT drug interaction. Management of side effects of ATT. Isoniazid preventive therapy for PLHIV. IRIS association with ART and ATT co-treatment and its management
STIs in PLHIVs	Screening and care of non-communicable diseases in children and adults
Importance of sexually transmitted infections in PHIV. Terms VDs, STDs, STIs, RTIs. STIs and reproductive tract infections. Classification of STIs, on an etiological basis and syndromic basis. Common symptoms and signs of STIs and epidemiology. How to assess risk for STI in men and women. Understand the relationship between HIV and STI and STI in PHIV. Importance of screening for STIs in PHIV (individual STIs to be screened and methods of screening). How to diagnose and provide evidence-based treatment of common STIs in PHIV on a syndromic basis	Understand the risk of non-communicable diseases in PLHIV. Screening and management of common NCDs in PHIV. Implementation of WHO Package of Essential Noncommunicable Diseases Interventions. Implementation of different components of care for children with HIV as recommended in consolidated guidelines. Identification of the non-communicable diseases in children and adults and timely reference to the subject experts
Infection prevention and control: laboratory safety, waste management	Data management of patients registered at the ART center and inventory/stock management
Understand the chain of infections. Hand washing steps, cough etiquette, sharp safety, safe injection practices, sterilization, and disinfection of patient care items and devices. Environmental infections prevention and control. Prevent the spread of pathogens and infection by using current techniques for hand hygiene and universal precautions. Protect yourself and those you serve by recognizing the chain of infections. How to identify, label, and dispose of hazardous waste. How to clean the facility - Guide and supervise cleaning/disinfection of facility	Maintenance of data on patients registered at the ART center manually and through MIS. Analyzation and reporting of patient data. Management of inventory/stock data manually and through LMIS. Understanding of different components of inventory management including procurement, storage, and distribution. Calculation and forecasting of consumption of ARV drugs, diagnostics, and consumables. Development of indent request. How to prevent stock-outs. How to avoid any stock losses/expiry

**Table 5:** Evaluation form used for participant's feedback regarding the workshop

Evaluation of three days training workshop for ART centers staff											
							Excellent	Very good	Good	Fair	Poor
Q.1	Overall how would you rate this three-day training workshop?										
Q.2	How were the trainers in inviting questions from participants?										
Q.3	How were the trainers responding to your questions?										
Q.4	How were the trainers responding to your questions?										
Q.5	How was the preparation of the trainers for conducting sessions?										
Q.6	How would you rate the training material of the workshop including Power-Point slides and overall training?										
Q.7	How would you rate the following aspects of the training venue of the workshop?										
							To great extent	To some extent	Did not meet expectations		
Q.8	Did this workshop meet your expectations?										
Q.9	Your suggestions for further improving the quality of workshop?										
